# Questionable Utility of Chloroform

**Published:** 1854-11

**Authors:** Robert Lee


### ON THE QUESTIONABLE UTILITY OF CHLOROFORM IN MIDWIFERY.

BY DR. ROBERT LEE, F. R. S.

(The Lancet, Dec. 24, 1853.)

Dr. Lee's paper, which was brought before the Royal Medical and Chirurgical Society, consists of an account of seventeen cases of parturition, in which chloroform had been inhaled with pernicious effects.

In these seventeen cases the author traces a series of injurious consequences to the employment of chloroform during labor.—Thus, in Cases 1 and 2, the contractions of the uterus were arrested by the chloroform, and delivery was completed by craniotomy. In Cases 3, 4, 5, 10, 14, 15, and 16, insanity and great disturbance of the brain followed its use. The necessity for delivery by the forceps was attributed to its employment in Cases 6, 8, 11, 12, and 13. Dangerous or fatal peritonitis or phlebitis ensued after the exhibition of chloroform in Cases 7, 8, 11, and 13. Epilepsy occurred in Case 14; and dangerous fits of syncope arose from its use in Case 17. The reports of friends had confided many more analogous cases, and public rumor swelled the list still further, but he was desirous of confining attention to those which came directly under his own observation. He thinks that a contemplation of the subtle action of this poison on the nervous system would have induced caution in its application to practice; but, on the contrary, the greatest levity had characterized its employment. Very soon after the discovery of its physiological effects, the author was astonished and confounded by the announcement of its application to widwifery; and it was not difficult for him to foresee that rashness in its application and use would lead to most deplorable results; and he regretted to find that in this he had been mistaken. It was not wonderful that women doomed to bring forth their offspring in pain and sorrow should seek to escape from the troubles of our race by means of this treacherous gift of science; neither could we feel surprise that the instances of women who were saved from the grievous pains of child-bearing, without bad consequences, should have for a time reduced to silence those unwelcome monitors who pointed to the possible evils of this new agent; but it did seem strange to the author that, amidst so wide-spread an experience us now existed of the noxious and dangerous effects of chloroform, it should be necessary for him to assemble the proofs of the havoc it had made. The two most serious effects produced by chloroform on women in labor were, a languid and deficient contraction of the uterus, and a greater susceptibility to the risks that arise from inflammation and fever. With regard to the first, the direct testimony of his own senses convinced him that the action of chloroform did very manifestly slacken the uterine contractions, and in some cases had put a stop to them altogether. Of the second class of effects, the risks of the puerperal condition were much complicated; for to inflammation and fever must be added severe cerebral and nervous disorders. He has no doubt that the use of this noxious agent ought to be expelled from the practice of midwifery. In conclusion, the author observes that, though his opinions had been confirmed by conversations with the most discreet and experienced practitioners, yet he entertained grave doubts of the result of the present appeal to the good sense of the profession, when he considered the arts used to propagate a faith in this practice. It had become almost an extra professional question. There was a systematic concealment of truth by physicians; appeals were made to the natural timidity of women, and the most fallacious promises of perfect safety were boldly held out. Conceited or ignorant women of fashion made a pastime of this as of other quackeries, and the cause of science and humanity was placed in the hands of the most presumptuous and frivolous part of the community, while young and inexperienced mothers were decoyed to their destruction. If he had helped to rescue the medical profession from the dominion of a great and dangerous error, and had placed some restraint on an ignominious and disgraceful practice, the author would rest satisfied that this essay had not been written in vain.

Various remarks having been made by different Fellows of the Society after the reading of the paper, Dr. Lee proceeded to reply to them.

He contended that there was no resemblance between a surgical operation and the process of natural labor. In natural labor, if the pains are strong and regular, women, in a vast majority of cases, are exposed to little danger, require no artificial assistance, and the function is only disturbed by interference. Mr. Ferguson had just stated that one of the principal benefits derived from chloroform in surgery is the great amount of muscular relaxation which it produces during operations. In midwifery this great amount of muscular relaxation would produce the most mischievous results; it would, in fact, induce partial or complete paralysis of the uterus, as in the case just related by Dr. Merriman, and in several of the cases detailed in the paper read that evening. A striking and fatal example of the same kind had occurred since the paper was presented to the Society. Only three drachms were used in the first stage of labor, but no proper contractions followed the expulsion of the fœtus, and the uterus remained uncontracted till the death of the patient some days after in convulsions. There were no symptoms of puerperal fever or local inflammation. "The uterus did not go down to the usual size, so much so as to give rise to the suspicion that there was another child, or some ovarian disease; but there was neither." Dr. Snow had related to the Society a case of arm presentation, where the uterus was completely paralyzed by the chloroform he exhibited, that Mr. French turned with great ease, the contractions being so violent that he had contemplated eviscerating the fœtus. Yet Dr. Snow affected to doubt whether chloroform and narcotic poisons impair the action of the uterus, and had expressed an opinion that in the cases he (Dr. Lee) had related in the paper, the sudden cessation of the uterine contractions after its use could not be referred to it. It was impossible to reconcile such contractions, but they admitted of a ready explanation. He lately perused a letter written by a fashionable lady, soon after her confinement, to a physician, which contained the following passage:—"*Chloroforme a la reine*, just a few drops on a handkerchief from time to time for the last hour; I found it a most indescribable alleviation, and that though never insensible." This was a correct account, he believed, of the way in which chloroform was administered by Dr. Snow in natural labor, and it would account very satisfactorily for his assertion that the uterine contractions were little, if at all, impaired by it. Fifteen drops sprinkled upon a handkerchief, and the lady now and then permitted to sniff a little of the vapor from the corner in the last hour of labor. If he (Dr. Lee) might be allowed in plain language to characterize this proceeding, he would say the whole was a mere pretence, and calculated only to deceive the weak, ignorant and credulous. The anæsthesia from chloroform, of which Mr. Ferguson had spoken, and which was usually, as we understood, the result in midwifery, was quite another affair from the *chloroforme a la reine* of Dr. Snow. Last week he saw a surgical operation performed upon a young woman, to whom six drachms had been administered. Her pupils were widely dilated, the breathing stertorious; there was foaming at the mouth; the pulse was rapid and feeble, and there was convulsive twitchings of the muscles of the extremities. No man in his senses would venture to reduce a woman to such a frightful condition in natural labor. In surgery it might be considered justifiable, but in midwifery it was wholly unjustifiable. Dr. Gream had stated that two ounces of chloroform might be given with safety in cases of natural labor; and though he admitted that it decidedly had the effect of diminishing the strength and regularity of the uterine contractions, yet still its influence might be so managed as to prevent the progress of labor being interfered with. If Dr. Gream would stand up in the face of the Society and state how chloroform could be so managed, he (Dr. Lee) would immediately sit down. (Dr. Gream here expressed his dissent; but Dr. Lee affirmed that this statement had been published by Dr. Gream in his pamphlet.) In forceps cases (continued Dr. Lee,) and in all the great operations of midwifery, chloroform could produce nothing but mischief; for in all these cases consciousness was the great safeguard of the patient. No forceps cases—and he had had as many as any one in that Society—were so unmanageable as those in which the consciousness was lost from puerperal convulsion, where the patient could not be held in the same position for any length of time. In uterine hemorrhage, and in all cases of protracted labor, from whatever cause, nothing but mischief could result from the use of that narcotic poison. The exhibition of chloroform in labor he held to be contrary to the second principles of physiology and morality. "In sorrow shalt thou bring forth children," was an established law of nature—an ordinance of the Almighty, as stated in the Bible, and it was in vain to attempt to abrogate the law. There could not be a doubt that it was a most unnatural practice to destroy the consciousness of women during labor, the pains and sorrows of which exerted a most powerful and salutary influence upon their religious and moral character, and upon all their future relations in life.—But he might put aside all these physiological and moral considerations, and rest his objection to the use of chloroform in labor upon the danger of introducing a subtle narcotic poison into the sytem at such a time. When only one drachm was given, who could be certain that it should not instantaneously be followed by the death of the person to whom it was administered? Upon this point the whole questions might be allowed to hinge.

